# Vitamin B12 deficiency in metformin-treated type-2 diabetes patients, prevalence and association with peripheral neuropathy

**DOI:** 10.1186/s40360-016-0088-3

**Published:** 2016-10-07

**Authors:** Marwan A. Ahmed, George Muntingh, Paul Rheeder

**Affiliations:** 1 Department of Pharmacology, Faculty of Health Sciences, University of Pretoria, Pretoria, South Africa; 2 Department of Internal Medicine, Steve Biko Academic Hospital, University of Pretoria, Pretoria, South Africa

**Keywords:** Diabetes, Metformin, Peripheral Neuropathy, Vitamin B12

## Abstract

**Background:**

The association between long-term metformin use and low vitamin B12 levels has been proven. However, the prevalence estimates of metformin-induced vitamin B12 deficiency showed considerable variation among the studies. The potential of the deficiency to cause or worsen peripheral neuropathy in type-2 diabetes mellitus (T2DM) patients has been investigated with conflicting results. The aim of the study was to investigate: 1) the prevalence of vitamin B12 deficiency in T2DM patients on metformin; 2) the association between vitamin B12 and peripheral neuropathy; 3) and the risk factors for vitamin B12 deficiency in these patients.

**Methods:**

In this cross-sectional study, consecutive metformin-treated T2DM patients attending diabetes clinics of two public hospitals in South Africa were approached for participation. Participation included measuring vitamin B12 levels and assessing peripheral neuropathy using Neuropathy Total Symptom Score-6 (NTSS-6) questionnaire. The prevalence of vitamin B12 deficiency (defined by concentrations <150 pmol/L) was determined. Those with NTSS-6 scores >6 were considered to have peripheral neuropathy. The relationship between vitamin B12 and peripheral neuropathy was investigated when the two variables were in the binary and continuous forms. Multiple logistic regression was used to determine risk factors for vitamin B12 deficiency.

**Results:**

Among 121 participants, the prevalence of vitamin B12 deficiency was 28.1 %. There was no difference in presence of neuropathy between those with normal and deficient vitamin levels (36.8 % vs. 32.3 %, *P =* 0.209). Vitamin B12 levels and NTSS-6 scores were not correlated (Spearman’s rho =0.056, *P =* 0.54). HbA1c (mmol/mol) (OR = 0.97, 95 % CI: 0.95 to 0.99, *P =* 0.003) and black race (OR = 0.34, 95 % CI: 0.13 to 0.92, *P =* 0.033) were risk factors significantly associated with vitamin B12 deficiency. Metformin daily dose (gram) showed borderline significance (OR = 1.96, 95 % CI: 0.99 to 3.88, *P =* 0.053).

**Conclusions:**

Close to third of metformin-treated T2DM patients had vitamin B12 deficiency. The deficiency was not associated with peripheral neuropathy. Black race was a protective factor for vitamin B12 deficiency.

## Background

Metformin is the cornerstone medication in the management of type 2 diabetes mellitus (T2DM) with estimates that it is routinely prescribed to 120 million patients with diabetes around the world [1]. Accumulating evidence from both observational and interventional studies has revealed the association between long-term use of metformin and vitamin B12 deficiency. It may be surprising knowing that the first article describing metformin-associated vitamin B12 malabsorption was published in 1971 [2]. While there is almost a current consensus on the medication’s potential to lower vitamin B12 levels, four decades have not been sufficient to clarify other significant aspects of the topic.

Despite the confirmed association between metformin and vitamin B12 deficiency, the real size of the problem is not yet properly quantified. Previous studies have shown that the prevalence of metformin-induced vitamin B12 deficiency varied greatly and ranged between 5.8 % and 52 % [3–11]. Such a wide range may be attributed to differences in cut-points chosen to define the deficiency, participants mean age, study settings, and metformin dose and duration of use.

Neuropathy may be the only clinical presentation of vitamin B12 deficiency, without haematological symptoms and signs [12]. The clinical picture of vitamin B12 deficiency has revealed salient alterations towards the predominance of neurological symptoms and signs over the last few decades [13]. Clinically, vitamin B12 deficiency peripheral neuropathy is indistinguishable from that of T2DM [14]. Vitamin B12 deficiency-associated peripheral neuropathy can also remain subclinical [13] and, possibly, interact with that of T2DM. Such neuropathy is thus likely to be misdiagnosed as diabetic neuropathy. The long-term use of metformin, mediated by vitamin B12 deficiency, may contribute to increasing the substantial burden of peripheral neuropathy in T2DM patients. Several studies have recently tried to clarify the possible link between long-term metformin use and its vitamin B12 deficiency-mediated peripheral neuropathy with conflicting results [7, 8, 14–16]. Research around the topic is usually faced by many challenges. Vitamin B12 minimal concentration requirements for proper neuronal functioning are not yet established [13]. Investigators are also challenged by the fact that neuropathy is a common complication of T2DM and its attribution to metformin-induced low vitamin B12 status can never be certain. Furthermore, it seems difficult to tackle the question through randomised controlled trials as the required study duration, sample size and ethical issues make the use of such designs impractical. All the currently available evidence comes from observational studies.

As the first study to explore the topic in African populations, the aims of our study were to determine the prevalence of vitamin B12 deficiency in metformin-treated T2DM patients, to investigate the relationship between vitamin B12 and peripheral neuropathy and to identify the risk factors for the vitamin deficiency in these patients.

## Methods

### Study design, patient recruitment and selection

This observational cross-sectional study was conducted at the diabetes clinics of departments of Internal Medicine at Steve Biko Academic Hospital and Kalafong Hospital in Pretoria, South Africa between August 2012 and January 2013. A consecutive sampling method was used. A provisional list of patients eligible to participate in the study was made from the records. On clinic days, all eligible patients were approached and asked to participate in the study. Those who accepted to participate signed the informed consent after reading the patient information leaflet.

Patients who met the following criteria were eligible to participate: willingness to participate in the study by signing the informed consent, diagnosed T2DM, use of metformin for six months or more and ability to read and write in English. Patients were excluded from the study if one or more of the following was present: pernicious anaemia, alcoholism, gastrectomy, gastric bypass surgery, pancreatic insufficiency, malabsorption syndromes, chronic giardiasis, surgery involving small intestine or HIV infection. Intake of vitamin B12 or any multivitamin preparation during the past 6 weeks, estimated Glomerular Filtration Rate (e-GFR) < 50 mL/min/1.73 m^2^ based on Cockcroft-Gault formula or diagnosed vitamin B12 deficiency were also exclusion criteria.

### Variables and data collection

The following information was obtained from participants’ records: age, sex, ethnic group, date of diagnosis of T2DM, most recent HbA1c (glycated hemoglobin) level, duration of metformin use, dosage of metformin, smoking status, eGFR, Body-Mass Index (BMI), use of gastric acid suppressants or low-dose acetylsalicylic acid (both were linked to vitamin B12 deficiency [17, 18]), coffee consumption, presence and history of other medical conditions and alcohol consumption or any substance abuse. Participants were considered alcohol users when they consumed one drink or more per day. Coffee consumption was defined as daily consumption of one cup or more.

Serum vitamin B12 was measured in Ampath Laboratories by UniCel DxI 800 instrument (Beckman Coulter, United States). Vitamin B12 deficiency was defined as levels <150 pmol/L [3, 5, 6, 8].

Neuropathy Total Symptom Score-6 (NTSS-6) questionnaire was used to grade peripheral neuropathy. The investigator asked each participant about frequency and intensity of six peripheral neuropathic symptoms, and used the answers to complete the questionnaire. The sum scores for each patient were obtained (possible range between 0.00 and 21.96). Grading the frequency and intensity of symptoms was based on the definitions of Bastyr et al. [19].

To reduce occurrence of bias, scoring of peripheral neuropathy was performed by the same investigator prior to accessing any sort of data that gives information on metformin dose and/or duration of use. Serum samples were sent for analysis after completing data extraction and peripheral neuropathy scoring.

### Statistical analysis

Sample size calculations showed that recruiting 120 participants would give a 7 % margin of error (95 % CI) around an estimated 20 % prevalence. STATA version 12 was used for statistical analysis. Two-sided P values ≤0.05 were considered statistically significant. Means (± standard deviations), actual numbers and percentages were used to describe data.

The prevalence of vitamin B12 deficiency was determined as the percentage of participants with vitamin levels <150 pmol/L. Relationship between vitamin B12 and peripheral neuropathy was examined by two methods. The first utilized chi-square test to investigate the relationship between the two binary variables created from the continuous variables of vitamin B12 (deficient for levels < 150 pmol/L, otherwise normal) and peripheral neuropathy (present for NTSS-6 scores > 6, otherwise absent). The second method used Spearman’s rank correlation coefficient (rho) to examine bivariate relationship between the two continuous variables of vitamin B12 levels and NTSS-6 scores.

Demographic and clinical characteristics of vitamin B12-deficient participants were compared to those with normal vitamin levels. Normality of continuous variables was examined by Shapiro-Wilk test. Unpaired t-test (for normally distributed data) and Mann–Whitney test (for non-normally distributed data) were used to compare the continuous variables. Chi-square test and Fisher’s exact test (when expected cell frequency was less than five) were used to compare categorical variables. Multiple logistic regression was performed where vitamin B12 status represented the binary dependent variable and all the variables with P values ≤0.25 in the univariate analysis were taken as independent variables. The variables metformin duration, metformin cumulative dose (product of total daily dose and duration of use) and T2DM duration were likely to be highly correlated. Three initial models, each contained one of these variables, were made to avoid the impact of the multicollinearity. Backwards stepwise manner was then used to reduce initial models to a final model, from which risk factors significantly associated with vitamin B12 deficiency were determined.

## Results

A total of 130 eligible patients were identified. Four patients did not agree to participate and 126 signed the informed consent. Of those who signed, 5 patients with eGFR values less than 50 mL/min/1.73 m^2^ were excluded. The final analysis was performed based on data obtained from 121 participants.

Table 1 shows participants’ demographic and clinical characteristics. Women represented 66 % of participants. The sample consisted of 89 (73.5 %) blacks, 19 (15.7 %) whites, 10 (8.3 %) Indians and 3 (2.5 %) coloured. Indian, coloured and white ethnic groups were all grouped together as non-black and represented 26.5 % of the sample. The sample mean age was 58.5 years and mean T2DM duration was 11.6 years. The means of metformin duration of use and total daily dose were 9.6 years and 2.4 g, respectively. The mean cumulative dose of metformin was 23.7 g. HbA1c, eGFR and BMI had mean values of 76 mmol/mol (9.1 %), 116.7 mL/min/1.73 m^2^ and 33.4 kg/ m^2^, respectively. Ninety six (79.3 %) participants were on insulin. Twelve (10 %) participants were on a PPI or H2RA while 92 (77.7 %) were on low-dose acetylsalicylic acid. The mean of serum vitamin B12 concentrations was 260.6 pmol/L and that of NTSS-6 scores was 5.11.

**Table 1:** Participants’ demographic and clinical characteristics (*n =* 121)

Characteristics	Values
Age (years)	58.5 ± 10.5
T2DM duration (years)	11.6 ± 7.5
Duration of metformin use (years)	9.6 ± 6.8
Total daily dose of metformin (gram)	2.4 ± 0.7
Cumulative dose of metformin (gram)	23.7 ± 18.2
eGFR (mL/min/1.73 m^2^)	116.7 ± 44.6
Women, n(%)	80(66)
HbA1c (mmol/mol) [%]	76 ± 27 [9.1 ± 2.5]
Smokers, n(%)	8(6.6)
Insulin use, n(%)	96(79.3)
Alcohol consumption, n(%)	4(3.3)
Coffee consumption, n(%)	23(19)
Race	
Black, n(%)	89(73.5)
Non-black, n(%)	32(26.5)
Number of daily metformin doses	
One, n(%)	3(2.5)
Two, n(%)	61(51.2)
Three, n(%)	55(46.2)
Acetylsalicylic acid use, n(%)	94(77.7)
PPIs or H2RAs use, n(%)	12(10)
BMI (kg/m^2^)	33.4 ± 6.3
Vitamin B12 levels (pmol/L)	260.6 ± 163.7
NTSS-6 Scores	5.11 ± 3.86

The data are shown as the means ± SD or n (%)

*BMI* Body-Mass Index, *eGFR* estimated glomerular filtration rate based on Cockcroft-Gault formula, *PPI* Proton Pump Inhibitor, *H2RA* Histamine 2 Receptor Antagonist, *HbA1c* Glycated haemoglobin, *NTSS-6* Neuropathy Total Symptom Score-6

Thirty four participants, representing 28.1 % of the sample, were vitamin B12-deficient.

**Fig. 1:**
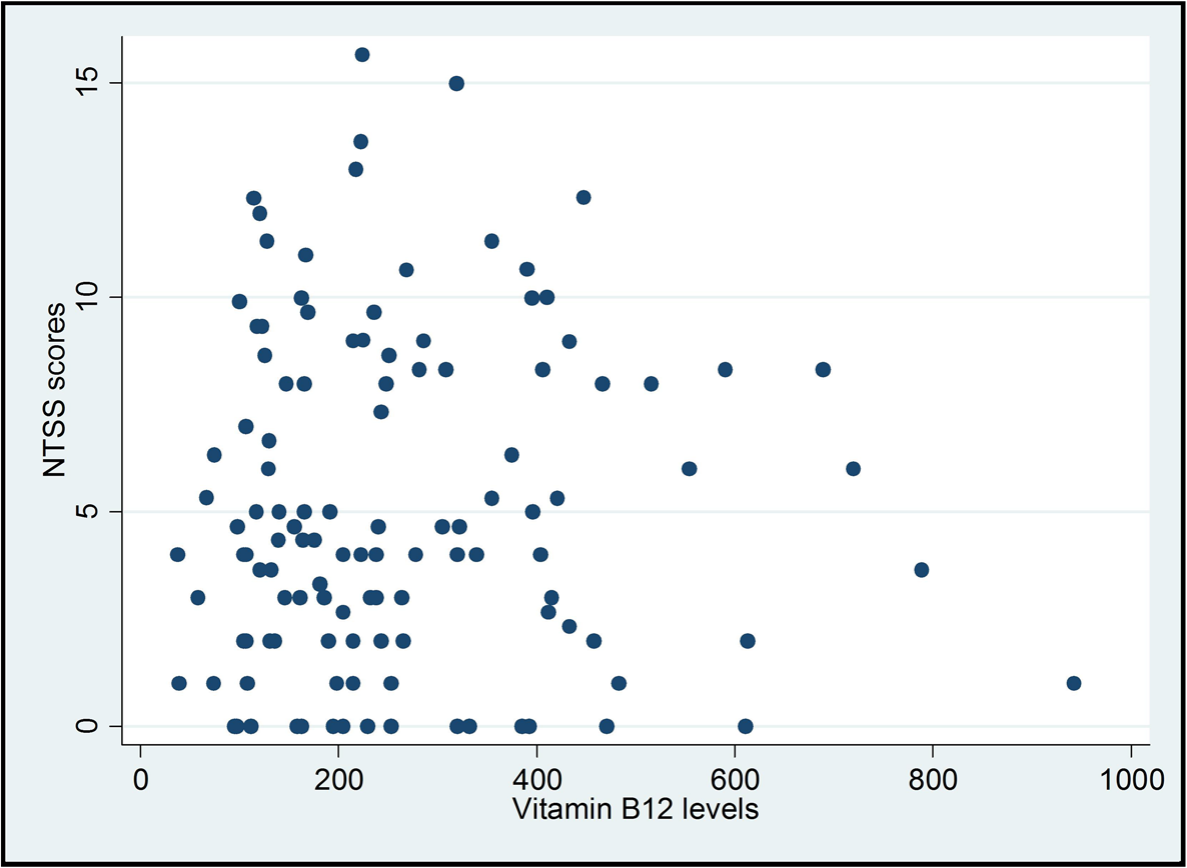
Scatter plot for vitamin B12 levels vs. NTSS-6 scores among all metformin-treated T2DM patients

**Fig. 2:**
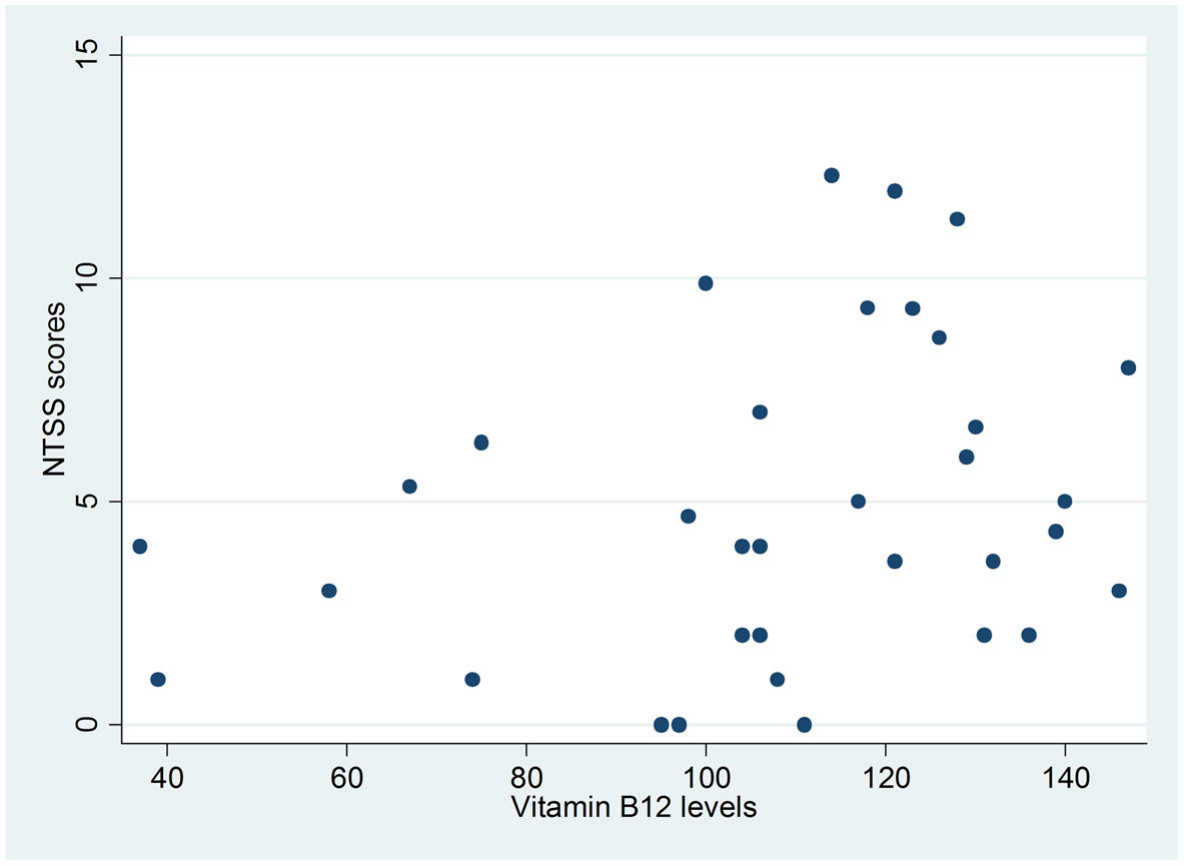
Scatter plot for vitamin B12 levels vs. NTSS-6 scores among metformin-treated T2DM patients with vitamin B12 deficiency

**Fig. 3:**
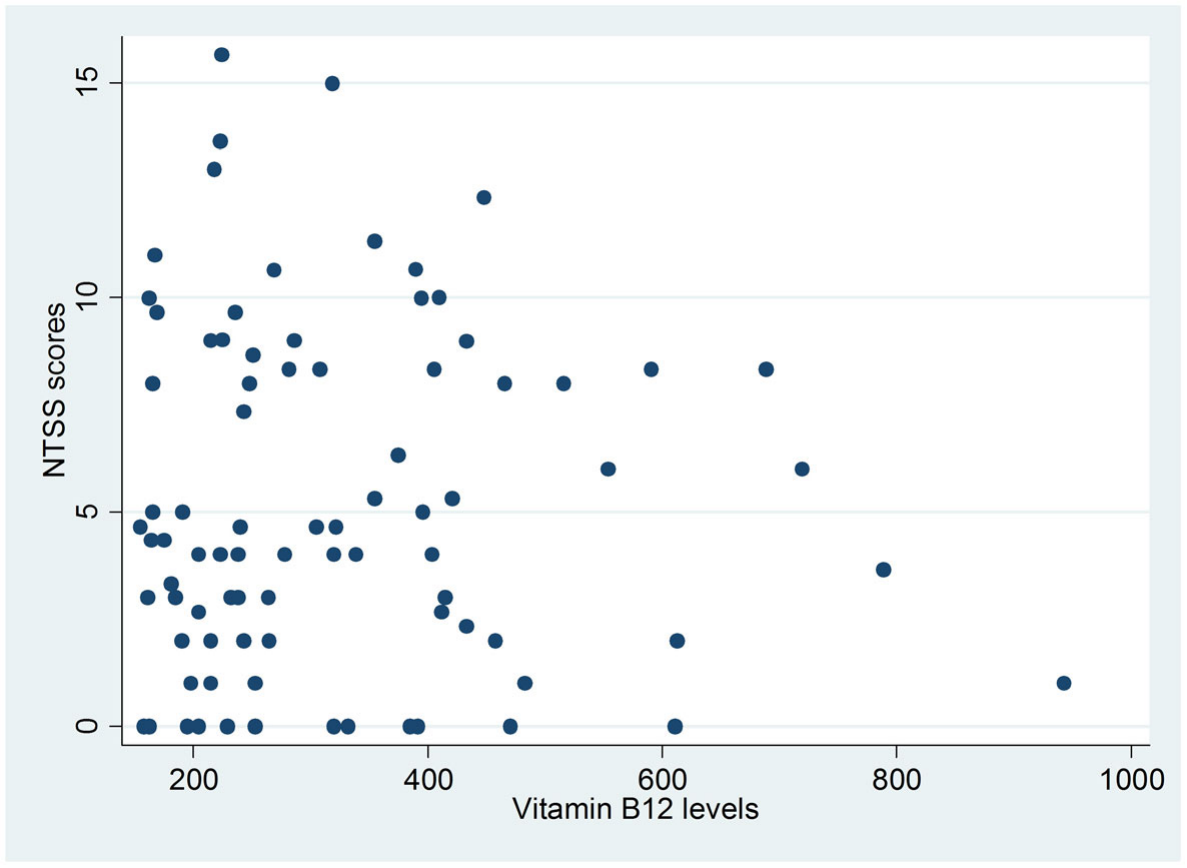
Scatter plot for vitamin B12 levels vs. NTSS-6 scores among metformin-treated T2DM patients with normal vitamin B12 levels

Table 2 shows that 32.3 % of vitamin B12 deficient participants had neuropathy compared to 36.8 % of those with normal vitamin levels. Chi square test results showed a Chi square statistic value of 0.209 with an associated probability of 0.647, indicating absence of enough evidence to claim an association between vitamin B12 status and neuropathy binary variables in the population. The value of Spearman’s rank correlation coefficient (rho) was 0.056 with a P value of 0.54, indicating that there was no sufficient evidence of association between vitamin B12 levels and NTSS-6 scores (Fig. 1). Comparable results of no association were also obtained when the correlation between vitamin B12 levels and NTSS-6 scores was examined in those with deficient (rho = 0.284, *P =* 0.10) (Fig. 2) and normal (rho = 0.057, *P =* 0.59) (Fig. 3) vitamin B12.

**Table 2:** Cross-tabulation of vitamin B12 status and peripheral neuropathy

	Peripheral Neuropathy		
Vitamin B12 status	Absent	Present	Total
Normal	55(63.2 %)	32(36.8 %)	87(100.00 %)
Deficient	23(67.7 %)	11(32.3 %)	34(100.00 %)
Total	78(64.46 %)	43(35.54 %)	121(100.00 %)

Univariate analysis (Table 3) revealed that vitamin B12-deficient participants were significantly older than those with normal vitamin levels (62.3 vs. 57 years, *P =* 0.012). They also had significantly longer metformin use duration (11 vs. 8 years, *P =* 0.015) and higher cumulative metformin dose (28.9 vs. 17 g, *P =* 0.009). Those with deficiency also revealed statistically significant lower HbA1c (57[7.4 %] vs. 79[9.4 %] mmol/mol, *P =* 0.001). Duration of T2DM was higher in vitamin-deficient participants with a P value that almost reached the statistical significance (12 vs. 9 years, *P =* 0.055).

**Table 3:** The characteristics of vitamin B12-deficient participants compared to those with normal vitamin B12 levels

Variable	Low vitamin B12 (*n =* 34)	Normal vitamin B12 (*n =* 87)	P value
Age (years)	62.3 ± 10.2	57.0 ± 10.2	0.012
T2DM duration (years)	12(8.75/17)	9(5/16)	0.055
Duration of metformin use (years)	11(6.75/13.25)	8(3/13)	0.015
Total daily dose of metformin (gram)	2.6 ± 0.7	2.4 ± 0.7	0.228
Cumulative dose of metformin (gram)	28.9(14.5/40.8)	17(7.7/31.3)	0.009
eGFR (mL/min/1.73 m^2^)	100.4(78.6/129)	108.5(88/150.7)	0.093
Sex			
Women, n(%) Men, n(%)	21(61.8) 13(38.2)	59(67.8) 28(32.2)	0.530
HbA1c (mmol/mol) HbA1c (%)	57(45/81) 7.4(6.3/9.6)	79(58/99) 9.4(7.5/11.2)	0.001
Insulin use, yes(%)	28(82.4)	68(78.2)	0.451
Acetylsalicylic acid use, yes(%)	30(88.2)	64(73.5)	0.081
Coffee use, yes(%)	9(26.4)	14(16)	0.191
Race			
Black, n(%) Non-black, n(%)	22(64.7) 12(35.3)	67(75.3) 20(24.7)	0.168
BMI (kg/m^2^)	34.0 ± 6.5	33.1 ± 6.3	0.469
Number of daily doses			
One, n(%) Two, n(%) Three, n(%)	0(0) 21(63.6) 12(36.6)	3(3.5) 40(46.5) 43(50)	0.198
Use of PPI or H2RA, yes(%)	5(14.7)	7(8)	0.271
NTSS scores	4.16(2/7.25)	4.33(2/8.33)	0.914

Data are expressed as mean ± standard deviation, median (25/75 percentile) or n (%)

Table 4 shows ORs, 95 % CIs and P values for vitamin B12 deficiency risk factors in the three initial models (A, B and C). The three models ultimately resulted in the same final reduced model (model D, Table 5). The final model has shown that the odds of having vitamin B12 deficiency was significantly reduced by being from black South African descent (OR = 0.34,95 % CI: 0.13 to 0.92, *P =* 0.033) and by increase in HbA1c value (mmol/mol) (OR = 0.97, 95 % CI: 0.95 to 0.99, *P =* 0.003). The model has also shown that metformin total daily dose (gram) increased the odds of having vitamin B12 deficiency with an approximately significant P value (OR = 1.96, 95 % CI: 0.99 to 3.88, *P =* 0.053).

**Table 4:** The initial multiple logistic regression models assessing independent predictors of vitamin B12 deficiency in metformin-treated T2DM participants

Independent variable	Model A		Model B		Model C	
	OR (95 % CIs)	P value	OR (95 % CIs)	P value	OR (95 % CIs)	P value
Metformin duration (years)	1.03 (0.96 to 1.10)	0.481	-	-	-	-
Cumulative metformin dose (g)	-	-	1.01 (0.98 to 1.04)	0.531	-	-
T2DM duration (years)	-	-	-	-	1.03 (0.97 to 1.10)	0.374
Total daily dose of metformin (g)	1.82 (0.87 to 3.80)	0.111	1.66 (0.71to 3.85)	0.239	1.82 (0.88 to 3.78)	0.107
Age (years)	1.03 (0.96 to 1.10)	0.416	1.03 (0.96 to 1.10)	0.423	1.03 (0.96 to 1.10)	0.429
HbA1c (mmol/mol)	0.98 (0.96 to 0.99)	0.034	0.97 (0.95 to 0.99)	0.036	0.98 (0.95 to 0.99)	0.034
Coffee Consumption (if yes)	1.82 (0.57 to 5.80)	0.310	1.81 (0.57 to 5.74)	0.315	1.86 (0.58 to 5.96)	0.294
Race (if black)	0.30 (0.10 to 0.88)	0.029	0.29 (0.10 to 0.87)	0.028	0.30 (0.10 to 0.89)	0.031
Acetylsalicylic acid use	2.64 (0.73 to 9.58)	0.140	2.63 (0.73 to 9.51)	0.141	2.61 (0.72 to 9.47)	0.144
Number of metformin daily doses	0.84 (0.33 to 2.11)	0.705	0.84 (0.33 to 2.12)	0.707	0.82 (0.32 to 2.06)	0.669
eGFR (mL/min/1.73 m^2^)	0.99 (0.98 to 1.01)	0.703	0.99 (0.98 to 1.01)	0.692	0.99 (0.98 to 1.01)	0.759

**Table 5:** The reduced multiple logistic regression model for risk factors of vitamin B12 deficiency in metformin-treated T2DM patients (Model D). OR > 1 indicates greater risk for vitamin B12 deficiency

Independent variable	Odds ratio (95 % CIs)	*P* value
Total daily dose of metformin (gram)	1.96 (0.99 to 3.88)	0.053
HbA1c (mmol/mol)	0.97 (0.95 to 0.99)	0.003
Race (if black)	0.34 (0.13 to 0.92)	0.033

## Discussion

### Prevalence of vitamin B12 deficiency

The present study has found that the prevalence of vitamin B12 deficiency, as defined by serum levels <150 pmol/L, among T2DM patients receiving metformin was 28.1 %. Comparing the obtained prevalence with the results of previous studies is not straightforward and should consider several factors. Table 6 shows the prevalence estimates and certain characteristics of the studies that used deficiency cutoff points ranging between 145 and 150 pmol/L. The table reveals study-related factors with potential to affect the obtained prevalence, including mean participants age, mean metformin daily dose, study settings, mean metformin duration of use and whether participants with renal impairment were excluded. Our reported prevalence is high relative to previously reported estimates. It is exactly the same as that of Beulens *et al.* study. Mean metformin dose and duration were higher in our study compared to Beulen *et al*’s. Their sample was, however, older. Such non-directional differences in variables make comparative speculations on factors behind the high prevalence of the vitamin deficiency in our sample an unattainable task. The present study had the highest means of metformin dose and duration of use, possibly explaining the high prevalence obtained.

**Table 6:** Characteristics of studies that measured the prevalence of metformin-induced vitamin B12 deficiency with diagnostic cut-points ranging between 145–150 pmol/L

Study	Number of metformin-treated patients	Prevalence	Mean age (years)	Mean metformin dose (gram)	Mean metformin duration of use (years)	Study setting	Exclusion of renally-impaired patients
De Jager et al. [3]	196	9.9 %^a^	64	2.1	4.3	Outpatient clinics of 3 non-academic hospitals, The Netherlands	Yes
Reinstatler et al. [4]	575	5.8 %	63.4	NA	5^b^	NHANES sample, United States	Yes
Hermann et al. [5]	53	8 %	58.5	2.2	5.2	Outpatient diabetes clinic of a general hospital, Sweden	Yes
Calvo Romero and Ramiro Lozano [9]	81	8.6 %	71.6	1.8	3.6	Internal medicine clinic of a first level hospital, Spain	No
Beulens et al. [11]	550	28.1 %	61.6	1.3	5.3	4 primary care centers, The Netherlands	No
De Groot-Kamphuis et al. [8]	164	14.1 %	62.6	-	4.9^b^	Secondary care outpatient diabetes clinic, The Netherlands	No
This study	121	28.1 %	58.5	2.4	9.6	Outpatient diabetes clinics of 2 tertiary hospitals, South Africa	Yes

*NHANES* National Health and Nutrition Examination Survey

^a^ Trial was analysed by Intention To Treat. Using Per Protocol analysis results in 14.5 % prevalence

^b^ Median value

### Relationship between vitamin B12 and peripheral neuropathy

The present study found no statistically significant difference in presence of neuropathy between those with normal and deficient vitamin levels (36.8 % vs. 32.3 %, *P =* 0.209). The levels of vitamin B12 and the NTSS-6 scores were not correlated (Spearman’s rho = 0.056, *P =* 0.54). Our results are in-line with those of a recently published study which reported that metformin use was not associated with the presence of diabetic peripheral neuropathy in T2DM patients [20]. Our findings are also in concordance with the results of the cross-sectional study of Chen et al. which revealed no significant differences between metformin users and non-users when neuropathy status was assessed by both objective (monofilament and neurothesiometry) and relatively subjective (questionnaires) measures [14]. Biemans et al. also reported no differences in occurrence of neuropathy in vitamin B12-deficient and –normal T2DM patients receiving metformin [15]. Our results are in contrast with the case–control study of Wile and Toth who reported more severe neuropathy among T2DM patients on metformin compared to non-metformin group [16]. Their results were obtained when the neuropathy severity was assessed by Toronto Clinical Scoring System (TCSS) and Neuropathy Impairment Score. The more objective electrophysiological tests showed no statistically significant differences among the two groups. Singh et al. also reported more severe neuropathy as assessed by TCSS in metformin-treated T2DM patients [7]. Our results also contrasts with the data of the cross-sectional study of de Groot-Kamphuis et al. who reported lower prevalence of neuropathy in metformin-treated T2DM patients compared to the non-metformin group [8].

Many animal studies have recently reported the glycemic control-independent neuroprotective impact of metformin. Mao-Ying *et al.* found that metformin reduced peripheral nerve endings loss and exerted protective effect against chemotherapy-induced peripheral neuropathy (CIPN) in mice [21]. Both peripheral neuropathy and CIPN share the glove-and-stocking distribution nature of sensory symptoms that includes parasthesia, dysthesia and pain [21]. Animal studies have also recently shown that metformin reversed induced neuropathic pain and protected against nerve injury [22], protected against neuronal apoptosis induced by ethanol [23], inhibited neuronal apoptosis in cortical cells [24], stimulated neurogenesis [25], and promoted neurogenesis following middle cerebral artery occlusion in mice [26]. Considering such impact of metformin, there can be two possible lines through which the medication affects the neuropathy status, excluding that related to glycemic control. One involves a positive impact through neuroprotective mechanisms, while the other induces neuropathy by enhancing vitamin B12 deficiency. Absence of association between vitamin B12 and peripheral neuropathy in our study may not thus totally preclude the potential of the medication to precipitate or worsen neuropathy through vitamin B12 deficiency. This theory may also explain the contradictory nature of results obtained by different studies.

Judicious interpretations of evidence around peripheral neuropathy as a clinical consequence of metformin-induced vitamin B12 deficiency may require considering certain medication-related features with possible significant methodological impacts. Being the cornerstone of the management of T2DM, it should be uncommon to encounter T2DM patients who are not on metformin. In observational studies that compare metformin users and non-users, T2DM patients who are not on metformin may hence be inherently different from those taking the medication. Being not on metformin is itself an abnormality with potential to make obtaining similar study groups a strenuous task. Having a control group may, therefore, negatively influence the validity in studies aiming at investigating the impact of metformin-induced vitamin B12 deficiency on neuropathy. The theory of the possible neuroprotective impact of metformin commented above may also lead to the same methodological conclusion. The possibility exists that neuroprotective effect of the medication group dilutes or predominates over its neuropathy-generating impact which is mediated by vitamin B12 deficiency. Comparing peripheral neuropathy in the control and metformin groups may thus produce distorted results that do not truly reflect the contribution of the metformin-induced vitamin B12 deficiency to the neuropathy status. From this perspective, designs that compare peripheral neuropathy among vitamin B12-deficient and –normal metformin-treated patients can theoretically result in more valid findings.

### Risk factors for vitamin B12 deficiency

Low HbA1c was a significant risk factor for vitamin B12 deficiency in the final (Table 5) (OR = 0.97, 95 % CI: 0.95 to 0.99, *P =* 0.003) as well as the three initial models (Table 4). Kang *et al.* reported, but did not explain, similar results [10]. They reported stronger association between HbA1c and vitamin B12 deficiency (OR = 0.74, CI: 0.56 to 0.99). Their study included T2DM patients who were either on metformin plus insulin or metformin plus sulfonylurea. Such stricter inclusion criterion may explain the difference in magnitude of OR between the present study and Kang et al’s. We think the association between HbA1c and vitamin B12 status can be, at least partly, explained by compliance to metformin treatment. The well-documented gastrointestinal adverse metformin reactions, adding to the high prevalence of gastrointestinal complications in diabetes patients, may propose higher risk of non-compliance among metformin users. Higher doses of metformin may result in more prominent gastrointestinal adverse reactions and higher rates of non-compliance. Our theory is that patients with poor glycemic control (higher HbA1c) may have poor compliance to metformin and thus higher vitamin B12 levels. Raw data from the present study revealed that 21.5 % of participants were on the maximum daily dose of metformin, possibly supporting our proposed theory on the interaction between dose, non-compliance, vitamin B12 levels and glycemic control. Considering the daily dose of metformin and renal function as direct explanatory factors of the relationship between HbA1c and vitamin B12 is statistically inappropriate as the relationship existed despite adjusting for metformin dose and eGFR (Table 4).

Black South African descent was a significant protective factor for vitamin B12 deficiency in the final model (OR = 0.34, 95 % CI: 0.13 to 0.92, *P =* 0.033) (Table 5) as well as the three initial models (Table 4). This is the first study to report ethnic differences in vitamin B12 levels among metformin-exposed T2DM patients. Reinstatler *et al.* found no statistically significant differences in vitamin levels among black, white and Hispanic metformin-treated patients in the United States [4]. Higher vitamin B12 levels in black compared to white general populations were previously reported [27, 28]. Higher levels of the vitamin binding proteins transcobalamin II and haptocorrin in black individuals were described in South African settings, and explained their relatively elevated vitamin B12 levels [29].

Daily dose of metformin has shown borderline significance as a risk factor for vitamin B12 deficiency in the final model (OR = 1.96, 95 % CI: 0.99 to 3.88, *P =* 0.053) (Table 5). The association between the medication daily dose and the deficiency is sensible, and was previously reported by multiple regression analyses of many studies [8, 11, 30].

### Limitations

Our study was conducted in tertiary academic specialist clinics, raising the possibility of over-representation of patients with complicated T2DM. Such patients may tend to have higher doses and durations of use of metformin. The study has only measured serum vitamin B12 levels to assess the vitamin status. Current recommendations suggest adding methylmalonic acid or homocysteine tests to better assess the intracellular status of the vitamin [31]. The absence of data on the compliance to metformin is also a limitation of this study. Compliance can have an impact on both the response to metformin and the levels of vitamin B12. To conclusively answer the secondary aim of investigating the relationship between peripheral neuropathy and vitamin B12 deficiency, a much larger sample size would have been needed to show a clinically important difference as small as 10 % to be statistically significant. NTSS-6 questionnaire, which is relatively subjective and symptom history-dependent, was the only used tool to assess neuropathy in the study. However, the questionnaire has been validated and was found suitable for evaluating peripheral neuropathy in clinical trials [19].

## Conclusions and recommendations

This is the first study to address the topic of metformin-associated vitamin B12 deficiency in African settings. The study demonstrated that the prevalence of vitamin B12 deficiency, defined by levels <150 pmol/L, in metformin-treated T2DM patients was as high as 28.1 %. There was no association between vitamin B12 and peripheral neuropathy. A novel finding was the association between black South African descent and lower odds of vitamin B12 deficiency in metformin-treated patients. We recommend regular screening for vitamin B12 deficiency in patients on long-term metformin.

As the first attempt to approach the topic in Africa, our study reveals the need for setting-specific evidence to tackle the subject. Further research that judiciously considers study design issues is warranted to clarify the possible impact of metformin-induced vitamin B12 deficiency on peripheral neuropathy in T2DM patients.

## Abbreviations

CIPN: Chemotherapy-Induced Peripheral Neuropathy
eGFR: Estimated Glomerular Filtration Rate
H2RAs: Histamine-2 Receptor Antagonists
HbA1c: Glycated hemoglobin
NHANES: National Health And Nutrition Examination Survey
NTSS-6: Neuropathy Total Symptom Score-6
PPIs: Proton Pump Inhibitors
T2DM: Type-2 Diabetes Mellitus
TCSS: Toronto Clinical Scoring System
